# Successful Removal of a Chronic Aspirated Foreign Body after Twelve Years

**DOI:** 10.1155/2018/8241591

**Published:** 2018-05-31

**Authors:** Radhika Z. Reddy, Yvonne M. Carter, David W. Hsia

**Affiliations:** ^1^VA Long Beach Medical Center, Department of Medicine, Division of Pulmonary and Critical Care Medicine, USA; ^2^Sinai Hospital of Baltimore, Department of Surgery, Division of Thoracic Surgery, USA; ^3^Harbor-UCLA Medical Center, Department of Medicine, Division of Respiratory and Critical Care Physiology and Medicine, USA

## Abstract

Chronic retention of aspirated foreign bodies is rare but can result in indolent systemic and respiratory symptoms. Bronchoscopy may show features of tissue reaction to the foreign body, including granulation tissue, endobronchial stenosis, strictures, edema, and airway distortion. The diagnosis of foreign body aspiration is often difficult to establish since some patients may not give a clear history of aspiration or may present late. In addition, patients may be misdiagnosed with chronic pneumonia, bronchitis, asthma, or malignancy. We present the case of a 42-year-old male who had a chronically retained piece of an aluminum beverage container in the left mainstem bronchus for 12 years. Careful history, radiographic evaluation, and bronchoscopic examination revealed the foreign body, which was successfully extracted by rigid bronchoscopy.

## 1. Case Presentation

A 42-year-old man presented with a nine-year history of intermittent productive cough. He also endorsed recurrent episodes of fevers, chills, and night sweats but denied shortness of breath, chest pain, hemoptysis, or weight loss. He presented to the Emergency Department for similar symptoms two months prior and was given a seven-day course of levofloxacin, with temporary improvement in symptoms. Past medical history was significant only for diabetes mellitus. He had no prior surgery and did not take any medications. He worked as a gardener and denied any history of smoking, alcohol, or drug use. On examination, vital signs were stable with normal oxygen saturation on room air. Chest auscultation revealed mildly decreased breath sounds and rhonchi in the left lower lung field. The remainder of the examination was normal.

**Figure 1:**
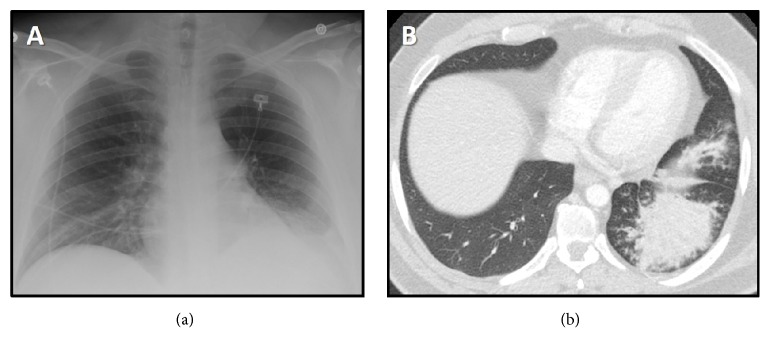
Chest radiograph (a) and computerized tomography (b) demonstrate left basilar lung opacification.

A chemistry panel and CBC were unremarkable, including a WBC of 7.0. Sputum AFB smears and bacterial cultures were all negative. Chest radiograph showed left basilar lung consolidation with tree-in-bud opacities and stenosis of the LMSB (Figure 1(a)). Chest CT scan showed extensive tree-in-bud opacities with confluent consolidation in the left lung base, enlarged hilar and mediastinal lymph nodes, and stenosis of the LMSB (Figure 1(b)).

**Figure 2:**
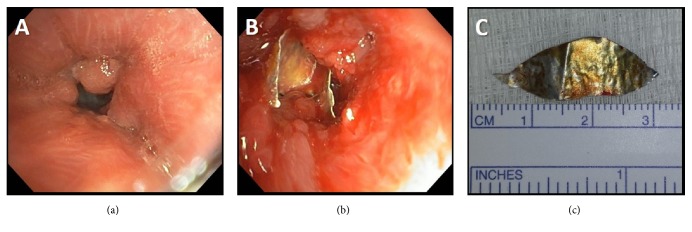
(a) Left mainstem bronchus with near-occlusion from obstructing granulation tissue. (b) Metallic foreign body appreciated in the distal left mainstem bronchus. (c) Metallic foreign body after removal.

Flexible bronchoscopy revealed near-occlusion and distortion of the mid-LMSB (Figure 2(a)). Mechanical debulking with endoscopic biopsy forceps allowed for passage of the bronchoscope to the distal LMSB where a metallic foreign body was appreciated (Figure 2(b)). Histologic analysis of the biopsied endobronchial tissue revealed inflammation and squamous metaplasia, but no evidence of malignancy.

Subsequently, the patient underwent rigid bronchoscopy and a 2.7 centimeter metal foreign body was retrieved using rigid bronchoscopy forceps (Figure 2(c)). Granulation tissue occluding the LMSB was treated with argon plasma coagulation. The patient reported immediate improvement in his symptoms. Upon further questioning, he recalled a previous aspiration of a small piece of an aluminum beverage container that he used in lieu of dental floss 12 years before. This object was retained over that time period until we removed it.

Follow-up surveillance bronchoscopy demonstrated persistent endobronchial stricture in the distal LMSB. Rigid bronchoscopy with radial incisions by electrocautery knife and balloon dilation was used to restore luminal patency. Repeat CT scan one month after endobronchial therapy showed resolution of the left lung tree-in-bud opacities and consolidation and restored patency of the LMSB. The patient's chronic cough resolved and he remains asymptomatic.

## 2. Discussion

Foreign body aspiration is uncommon in healthy adults and occurs more commonly in children and in the elderly [1]. In adults, the incidence of foreign body aspiration peaks in the sixth decade of life. Adults who present with foreign body aspiration often have an underlying risk factor such as neuromuscular disease, altered mental status, head or facial trauma, endotracheal intubation, dental procedures, underlying pulmonary disease, or sedative or alcohol use [2]. In addition, adults with an impaired swallowing reflex due to underlying medical conditions are also at higher risk of foreign body aspiration [2]. Commonly aspirated foreign bodies include metallic objects (pins, screws, and nails), organic objects including food particles, plastic objects, and teeth [3].

Foreign body aspiration can present as a life-threatening emergency with acute airway obstruction and respiratory failure requiring urgent intervention; however, most patients present with indolent chronic symptoms [4]. Chronic cough is the most common symptom and is present in two-thirds of patients. Other commonly reported symptoms include hemoptysis, fever, and dyspnea [4]. Radiographic features of foreign body aspiration include nonresolving pneumonia, atelectasis, unilateral hyperinflation, and localized bronchiectasis [5]. If the foreign body is radiopaque, it can be directly visualized on chest X-ray. However, it may be obscured by chronic parenchymal changes [5].

Bronchoscopic evaluation of the airways may directly visualize the foreign body if it was recently aspirated [6]. However, in the case of chronic aspiration, bronchoscopy may show features of tissue reaction to the foreign body, including granulation tissue, endobronchial stenosis, strictures, edema, and airway distortion [4, 6]. In one series of patients undergoing bronchoscopy for suspicion of foreign body aspiration, the foreign body was encountered in 49 of the 65 patients (75%) [3]. Other bronchoscopic findings included granulation tissue, mucosal edema, and bronchial stenosis. The right lower lobe bronchus (30%) and left mainstem bronchus (20%) were the most common locations of foreign bodies [3].

The diagnosis of foreign body aspiration is often difficult to establish in patients for several reasons [7]. Some may not give a clear history of aspiration or may present late, up to months to years after the initial aspiration event [8]. In one study, only 25% of patients presented within 7 days of the aspiration event [3]. In some patients, the clinical symptoms may be subtle and remain undetected for years, especially if a small foreign body lodges in lobar or segmental bronchi [9]. One case report describes aspirated* Coptis chinensis*, a Chinese herbal medicine, for 10 years [10]. Another case series described a piece of metal in a patient's right lung for 27 years following an industrial accident [11], while the longest described foreign body retention is 40 years [2]. These cases of chronic retention of foreign bodies in the airways are very rare, and our patient is amongst the few described extending beyond a decade.

Occult foreign bodies may also be discovered incidentally when bronchoscopy is performed to evaluate a chronic cough, hemoptysis, or nonresolving pneumonia [12]. In addition, patients may be misdiagnosed with chronic pneumonia, bronchitis, asthma, or malignancy. Therefore, a recurrent pneumonia that fails to respond to antimicrobial therapy should prompt bronchoscopic examination for evaluation of obstruction by a foreign body or tumor [13].

Definitive treatment of foreign body aspiration requires removal of the foreign body. The first step in management of suspected foreign body aspiration in adults is a flexible bronchoscopy [14]. Extraction of the foreign body by flexible bronchoscopy is successful in approximately 90% of patients [3]. The advantages of initial flexible bronchoscopy include cost effectiveness and the ability to be performed as an outpatient [14]. Rigid bronchoscopy is pursued in cases where flexible bronchoscopy is unsuccessful or inadequate for safe extraction and simultaneous airway management [14]. In addition, if foreign bodies are impacted by significant granulation tissue or are difficult to grasp with flexible forceps due to size or shape, rigid bronchoscopy should be used for extraction. Endobronchial ablation, cryotherapy, or airway dilation techniques may be necessary in cases where foreign body retention has caused significant granulation tissue or airway stenosis [15]. Our case demonstrates that successful bronchoscopic removal may still be possible despite airway changes that occurred secondary to the aspirated foreign body over a 12-year period.

## Abbreviations

AFB: Acid fast bacilli
CBC: Complete blood count
CT: Computed tomography
CXR: Chest radiograph
LMSB: Left mainstem bronchus
WBC: White blood count.
